# Changes in HIV Pre-exposure Prophylaxis (PrEP) Coverage at State and County Level During the COVID-19 Pandemic in the United States

**DOI:** 10.1007/s10461-023-04180-3

**Published:** 2023-09-26

**Authors:** Mahdi Fallahi, Jenny S. Guadamuz, Andrew Shooshtari, Dima M Qato

**Affiliations:** 1 Program on Medicines and Public Health, Titus Family Department of Clinical Pharmacy, Alfred E. Mann School of Pharmacy and Pharmaceutical Sciences, University of Southern California, Los Angeles, CA USA; 2 Division of Health Policy and Management, University of California, Berkeley School of Public Health, Berkeley, CA, USA; 3 Leonard D. Schaeffer Center for Health Policy and Economics, University of Southern California, Los Angeles, CA USA; 4 Spatial Sciences Institute, University of Southern California, Los Angeles, CA USA

**Keywords:** HIV, Prevention, Disparities

## Abstract

This study quantifies the prevalence and trends in weekly PrEP coverage at the national, state and county-level, before and during the COVID-19 pandemic in the United States.We estimated weekly PrEP coverage using longitudinal individual-level pharmacy claims from IQVIA LRx for a cohort of PrEP users (N = 287,493) ages 16 to 85 years between December 29th, 2019 and November 8th, 2020. Weekly PrEP coverage was defined as PrEP use among individuals at high risk for HIV. We conducted an interrupted time series analysis to quantify changes in weekly PrEP coverage before (December 29th, 2019 - March 8th, 2020) and during (March 29th - November 8th, 2020) the COVID-19 pandemic at the national, state and county-level by county characteristics, specifically by EHE priority jurisdiction, racial/ethnic composition, and urbanity. Nationally, weekly PrEP coverage among individuals ages 16 to 85 at high risk for HIV declined by 11.5% (from 11.0% before to 9.5% during the pandemic; t = 8.02,p < 0.01). Weekly PrEP coverage declined in all states and most counties yet varied substantially across states and counties. Geographic disparities in weekly PrEP coverage were also observed between urban EHE priority counties with significantly lower rates in counties with ≥ 50% Black/Latinx population when compared to their counterparts (7.9% vs. 11.2%; t = 18.91,p < 0.01);these disparities were most pronounced in California and New York. Weekly PrEP coverage was much lower than the 25% annual coverage reported by the Centers for Disease Control and geographic disparities observed within states likely contribute to the persistent racial/ethnic disparities in new HIV diagnoses observed within those states.

**Supplementary Information:**

The online version contains supplementary material available at 10.1007/s10461-023-04180-3.

## Introduction

The Department of Health and Human Services aims to increase pre-exposure prophylaxis (PrEP) coverage to more than 50% through the Ending the HIV Epidemic (EHE) initiative [1]. To target populations most at risk for HIV, the EHE initiative has identified 48 urban counties, Washington D.C., and San Juan, Puerto Rico, that account for more than half of new HIV diagnoses, and seven states where rural populations are disproportionately at-risk as EHE priority jurisdictions in the U.S. [2]. Despite these efforts to expand PrEP coverage, only 1 in 4 persons at high risk for HIV used this safe and effective preventive medication in 2020, with persistent geographic and racial/ethnic disparities in annual PrEP coverage across states and counties in the U.S. [3]. In fact, Black and Hispanic/Latinx populations persistently account for more than 75% of new HIV diagnoses and less than 20% of PrEP users in the U.S. [4].

Despite these insights on annual PrEP coverage, information on weekly PrEP coverage at the national, state, and county-level, including by race/ethnicity, is not available. Weekly (vs. annual) PrEP coverage is important to monitor because PrEP adherence is critical to its effectiveness in the prevention of HIV transmission [5]. In this study, we quantify weekly PrEP coverage at the national, state, and county-level before and during the COVID-19 pandemic. We hypothesize that weekly PrEP coverage is lower than annual coverage because ~ 50% of persons that initiate PrEP discontinue within 1-year, [6] and these coverage rates are lowest in disproportionately Black and Hispanic/Latinx counties due to known disparities by race/ethnicity^3^.

## Methods

### Data Sources

We used individual-level pharmacy claims data from IQVIA Real World Longitudinal Prescription Claims (IQVIA LRx) to identify PrEP users (N = 287,493) ages 16 to 85 years between December 29th, 2019 and November 8th, 2020 in the U.S. The IQVIA LRx is a longitudinal database that includes patients’ prescriptions followed across years, retail and non-retail pharmacies, and payment sources. The dataset captures more than 90% of prescriptions from retail pharmacies, 70% of mail service pharmacies, and 70% of pharmacies in long-term care facilities in the U.S. [7]. We identified PrEP prescriptions in the IQVIA LRx that included tenofovir disoproxil fumarate and emtricitabine (FTC/TDF) and tenofovir alafenamide and emtricitabine (FTC/TAF).

HIV Surveillance data from the CDC was used to identify the total population at high risk of HIV for which PrEP is indicated at the national, state, and county-level [2, 4]. The population at high risk of HIV was defined as the number of persons with indications for PrEP [1].

We defined counties as EHE priority counties based on the EHE priority designation which includes 48 urban counties, Washington D.C., and 20 counties in the 7 priority states with substantial burden of HIV in rural areas (Alabama, Arkansas, Kentucky, Mississippi, Missouri, Oklahoma, and South Carolina) [3]. San Juan was excluded from analysis due to insufficient data regarding the number of PrEP users.

Urbanization status was defined as rural or urban, following the 2013 National Center for Health Statistics’ (NCHS) Urban–Rural Classification Scheme for Counties. Counties were defined as urban if they were located in large central metro, large fringe metro, medium metro, and small metro areas [6]. All other counties were defined as rural. Our analyses included 3,136 counties that consist of 1,163 (37.09%) urban and 1,973 (62.91%) rural counties. We also characterized counties based on having populations whose combined Black and Hispanic/Latinx populations were ≥ 50% or < 50% of the total population.

### Outcome Measure

Our primary outcome measure was weekly PrEP coverage, defined as PrEP use among the total population at high risk for HIV at the national, state, and county-level [2]. As recommended by the CDC, PrEP is most effective when it is taken consistently every day. Taking at least a one-day supply of PrEP can significantly reduce the risk of HIV transmission [8]. PrEP use was defined as at least a one-day supply of PrEP for each week based on continuing or new prescriptions filled at retail pharmacies. To identify the total population at high risk for HIV, we utilized the annual estimated number of individuals with indications for PrEP as denominator. Then, we divided the number of individuals who were prescribed PrEP during the given week by the corresponding total population at high risk for HIV.

Data on the dates of prescription dispensing were converted to weekly data, with weeks starting on Sunday and ending on a Saturday. This study included PrEP coverage over a span of 46 weeks between December 29, 2019, and November 8, 2020. We assessed the general trends of weekly PrEP coverage at the national, state, and EHE priority county-level before and during COVID-19.

### Statistical Analysis

We conducted an interrupted time series analysis using segmented regression to estimate weekly PrEP coverage before (December 29th, 2019, - March 8th^th^, 2020) and during (March 29th - November 8th, 2020) the COVID-19 pandemic at the national, state, and the county-level by county characteristics, specifically by EHE priority status,, racial/ethnic composition, and urbanity. This study used de-identified data and was considered exempt by the institutional review board at the University of Southern California. Analyses were conducted using Stata statistical software, version 16.1 (Stata Corp, LLC).

## Results

**Fig. 1:**
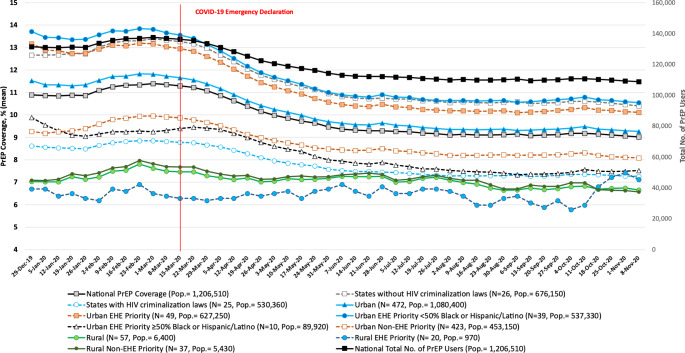
Trends in Weekly PrEP Coverage Nationally and by EHE Priority Status in Urban and Rural Counties in United States, 2020. **Note**: Phase 1 of the Ending the HIV Epidemic initiative focuses on 48 urban counties, Washington D.C., and San Juan, Puerto Rico, where more than 50% of HIV diagnoses occurred, and 7 states with substantial burden of HIV in rural areas that include Alabama, Arkansas, Kentucky, Mississippi, Missouri, Oklahoma, and South Carolina. San Juan is excluded from analysis due to insufficient data regarding the number of PrEP users. Rural counties in these 7 states were defined as rural EHE priority counties. Urbanization status was defined as rural or urban following the 2013 National Center for Health Statistics’ (NCHS) Urban–Rural Classification Scheme for Counties. Counties are defined urban if located in large central metro, large fringe metro, medium metro, and small metro. N denotes number of counties and Pop refers to population at risk for HIV for which PrEP is indicated. States with HIV-specific criminal laws include Arkansas, Delaware, Florida, Georgia, Idaho, Indiana, Iowa, Kentucky, Louisiana, Maryland, Michigan, Mississippi, Missouri, Nebraska, Nevada, North Carolina, North Dakota, Ohio, Oklahoma, Pennsylvania, South Carolina, South Dakota, Tennessee, Utah, and Washington

Nationally, the average weekly PrEP coverage among individuals ages 16 to 85 at high risk for HIV declined by 11.5% from 11.0% before (95% CI, 10.9-11.1%) to 9.5% (95% CI, 9.3-9.7%) during the pandemic (Fig. 1 and eTable 1). Declines in weekly PrEP coverage were only observed in urban (vs. rural) EHE priority counties. During the pandemic, PrEP coverage in EHE priority rural counties was considerably less than the national average (6.5% vs. 9.5%; t = 26.01, p < 0.01). Similar to before, during the pandemic weekly PrEP coverage was persistently higher in urban when compared to rural counties, (9.8% vs. 7.0%; t = 25.39, p < 0.01), including in EHE priority (10.7% vs. 6.5%; t = 28.01, p < 0.01) and non-EHE priority counties (8.5% vs. 7.1%; t = 19.57, p < 0.01). However, among urban EHE priority counties, weekly PrEP coverage was significantly lower in counties with ≥ 50% Black/Latinx population when compared to their counterparts (7.9% vs. 11.2%; t = 18.91, p < 0.01).

**Fig. 2:**
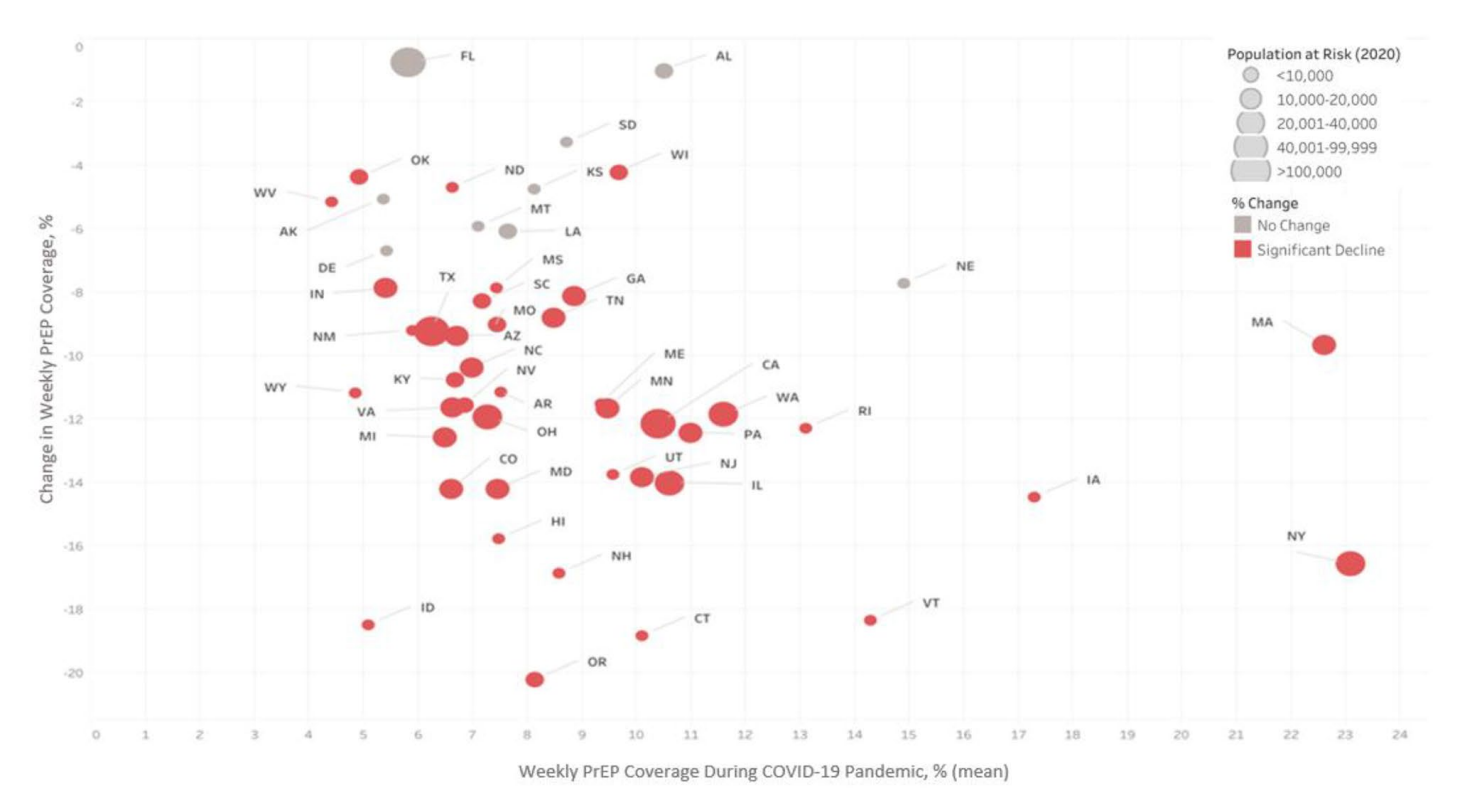
Changes in Weekly PrEP Coverage at the State-Level During the COVID-19 Pandemic in the U.S. ^a^ Before pandemic period is defined the week of December 29,2019, through the week of March 8,2020. The during pandemic period is defined the week of March 29, 2020, through the week of November 8, 2020. Intervention week is March 29, 2020. ^b^ For estimates of mean weekly PrEP coverage, predicted levels of PrEP coverage are calculated from a linear regression of the weekly PrEP coverage on the weekly trends before and during the pandemic for each state and county. ^c^ % change is calculated as: Change in Level/(Level Before Pandemic) ×100. Change in level is the coefficient of immediate change in the PrEP coverage in the interrupted time series analysis. ^d^ Change in level of weekly PrEP coverage statistically significant at p<0.05. All statistically significant differences observed were declines (vs. increases) in PrEP coverage.

Although weekly PrEP coverage declined in all states, throughout the pandemic (March 29th - November 8th, 2020) weekly PrEP coverage varied substantially across states with the highest rate observed in New York (23.1%) and Massachusetts (22.6%). Of the seven rural priority states, Alabama had the highest weekly PrEP coverage (10%) and Oklahoma lowest (5%) (Fig. 2 and eTable 2). Among the five states that account for more than half of persons at high risk for HIV, PrEP coverage was highest in New York (23.1%), followed by Illinois (10.7%) and California (10.5%) and was substantially lower in Texas (6.3%) and Florida (5.8%).

**Fig. 3:**
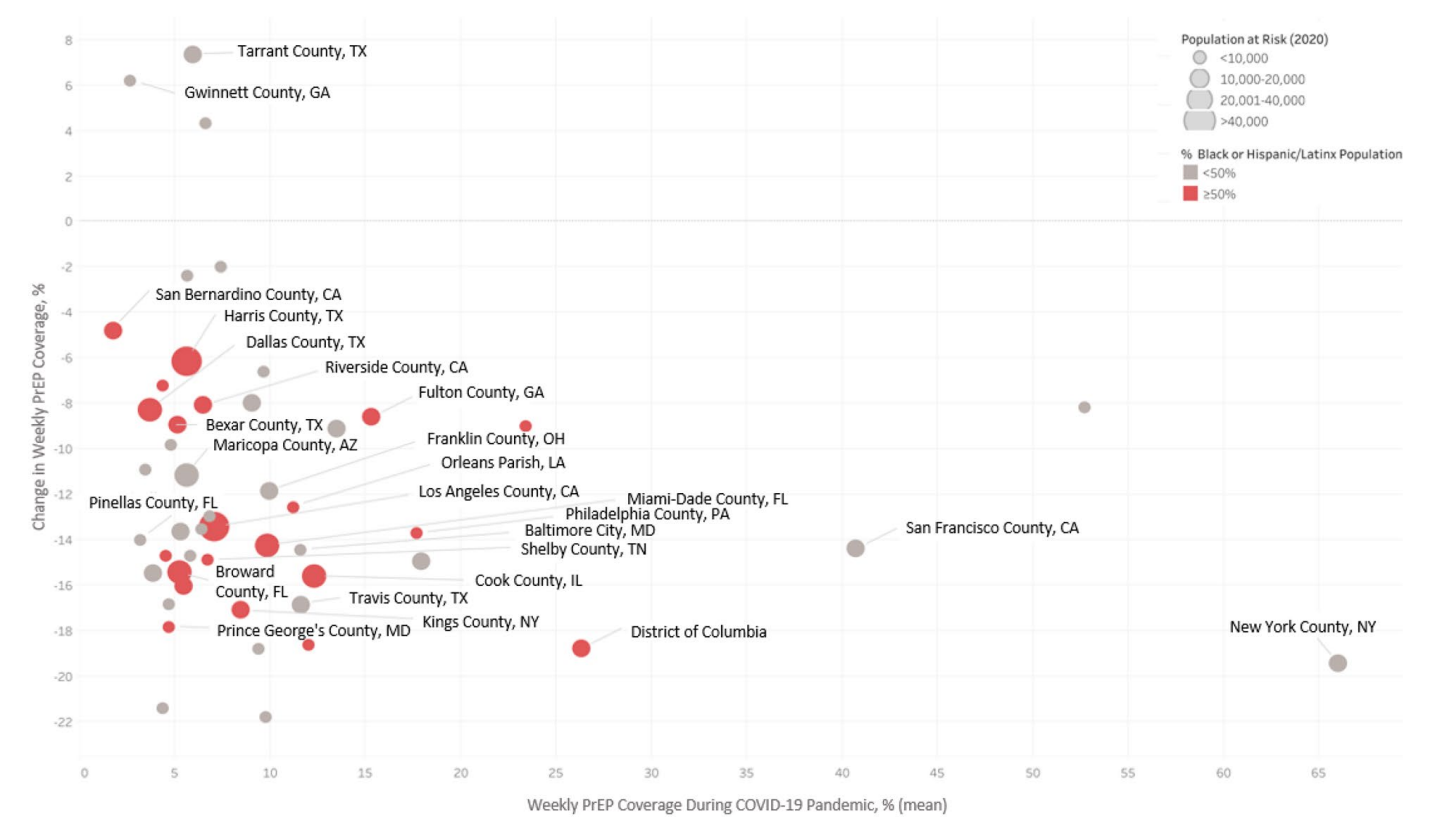
Changes in Weekly PrEP Coverage in Urban Ending the HIV Epidemic (EHE) Priority Counties During the COVID-19 Pandemic in the U.S. ^a^ Before pandemic period is defined the week of December 29,2019, through the week of March 8,2020. The during pandemic period is defined the week of March 29, 2020, through the week of November 8, 2020. Intervention week is March 29, 2020. ^b^ For estimates of mean weekly PrEP coverage, predicted levels of PrEP coverage are calculated from a linear regression of the weekly PrEP coverage on the weekly trends before and during the pandemic for each state and county. ^c^ % change is calculated as: Change in Level/(Level Before Pandemic) ×100. Change in level is the coefficient of immediate change in the PrEP coverage in the interrupted time series analysis. ^d^ Change in level of weekly PrEP coverage statistically significant at p<0.05. All statistically significant differences observed were declines (vs. increases) in PrEP coverage.

Urban EHE priority counties had the highest weekly PrEP coverage throughout 2020, yet declines during the pandemic, which averaged − 12.6% (eTable 1), were most pronounced in Queens County, New York (-21.8%), Hamilton County, Ohio (-21.4%), and New York County, New York (-19.5%) (Fig. 3 and eTable 3). Geographic disparities in weekly PrEP coverage were observed between urban EHE priority counties, including within a specific state, and were most pronounced in California and New York. Specifically, weekly PrEP coverage in San Francisco County (35.9%) was 6-times greater than Los Angeles County (6.3%) and New York County (61.0%) 8-times greater than Kings County (7.9%) during the pandemic **(**Fig. 2 and eTable 3). San Francisco County and New York County have < 50% Black or Latinx population, whereas in Los Angeles County and Kings County ≥ 50% of the population is Black/Latinx.

## Discussion

In this study, we found that weekly PrEP use declined during the COVID-19 pandemic in 2020, with only 1 in 12 persons at high risk for HIV taking PrEP at least once a week by November 2020 in the U.S. These findings indicate that weekly PrEP coverage is much lower than the 25% annual coverage reported by the CDC, [2] and are consistent with evidence that many PrEP users that initiate treatment discontinue within 1- year. [5].

Weekly PrEP coverage in 2020 varied by the urbanization status of the EHE priority counties, with rural counties having significantly lower rates than the national average. Our findings that the lowest weekly PrEP coverage rates were observed in disproportionately Black or Hispanic/Latinx urban EHE priority counties in a specific state, such as Los Angeles County and Kings County, suggest these geographic disparities likely contribute to persistent racial/ethnic disparities in new HIV diagnoses observed in that state [6]. Future efforts should monitor weekly PrEP coverage to better evaluate equity in PrEP adherence and target Black/Latinx communities at highest risk of PrEP discontinuation to reduce geographic disparities in HIV in the U.S.

### Electronic Supplementary Material

Below is the link to the electronic supplementary material.

Supplementary Material 1
